# Genome constellations of 24 porcine rotavirus group A strains circulating on commercial Thai swine farms between 2011 and 2016

**DOI:** 10.1371/journal.pone.0211002

**Published:** 2019-01-23

**Authors:** Supansa Tuanthap, Sompong Vongpunsawad, Supol Luengyosluechakul, Phanlert Sakkaew, Apiradee Theamboonlers, Alongkorn Amonsin, Yong Poovorawan

**Affiliations:** 1 Interdisciplinary Program of Biomedical Sciences, Graduate School, Chulalongkorn University, Bangkok, Thailand; 2 Center of Excellence in Clinical Virology, Faculty of Medicine, Chulalongkorn University, Bangkok, Thailand; 3 Department of Veterinary Medicine, Faculty of Veterinary Science, Chulalongkorn University, Bangkok, Thailand; 4 Department of Veterinary Public Health, Faculty of Veterinary Science, Chulalongkorn University, Bangkok, Thailand; 5 Center of Excellence for Emerging and Reemerging Infectious Diseases in Animals, Faculty of Veterinary Science, Chulalongkorn University, Bangkok, Thailand; Universidad de la Republica Uruguay- CENUR Litoral Norte Sede Salto, URUGUAY

## Abstract

Rotavirus A (RVA) infection is a major cause of diarrhea-related illness in young children. RVA is also one of the most common enteric viruses detected on pig farms and contributes to substantial morbidity and mortality in piglets. Long-term multi-site surveillance of RVA on Thai swine farms to determine the diversity of RVA strains in circulation is currently lacking. In this study, we characterized the 11 segments of the RVA genome from 24 Thai porcine RVA strains circulating between 2011 and 2016. We identified G9 (15/24) and P[13] (12/24) as the dominant genotypes. The dominant G and P combinations were G9P[13] (n = 6), G9P[23] (n = 6), G3P[13] (n = 5), G9P[19] (n = 3), G4P[6] (n = 2), G4P[19] (n = 1), and G5P[13] (n = 1). Genome constellation of the Thai strains showed the predominance of Wa-like genotype (Gx-P[x]-I1/I5-R1-C1-M1-A8-N1-T1/T7-E1/E9-H1) with evidence of reassortment between the porcine and human RVA strains (e.g., G4-P[6]-I1-R1-C1-M1-A8-N1-T1-E1-H1 and G9-P[19]-I5-R1-C1-M1-A8-N1-T7-E9-H1). To assess the potential effectiveness of rotavirus vaccination, the Thai RVA strains were compared to the RVA strains represented in the swine rotavirus vaccine, which showed residue variations in the antigenic epitope on VP7 and shared amino acid identity below 90% for G4 and G5 strain. Several previous studies suggested these variations might effect on virus neutralization specificity and vaccine efficacy. Our study illustrates the importance of RVA surveillance beyond the G/P genotyping on commercial swine farms, which is crucial for controlling viral transmission.

## Introduction

Rotavirus is highly contagious and is frequently responsible for acute gastroenteritis in humans and animals. It is a major cause of diarrhea-associated childhood hospitalization for children younger than 5 years of age [1]. On swine farms, rotavirus A (RVA) infection contributes to a substantial economic loss [2]. Complicating management and control of RVA infection are an unpredictable pattern of outbreaks, unknown passive immunity within the nursing herds, feasibility of mass vaccination, and co-infection with other enteric viral pathogens. Therefore, RVA infection remains an important threat to the pig industry.

Rotavirus is a member of the family *Reoviridae* and the genus *Rotavirus* [3]. Viral genome is approximately 18.5 kilobase pairs and consists of 11 double-stranded RNA segments. The virion forms triple concentric capsid layers of six structural proteins (VP1 to VP4, VP6, and VP7) and six non-structural proteins (NSP1 to NSP6). The outer capsid layer is formed by the glycoprotein VP7 and the protease-sensitive VP4, of which their nucleotide sequences (G and P genotypes, respectively) form the basis of a binary classification system for RVA strains. VP6 forms the intermediate layer, and VP1, VP2, and VP3 proteins and the non-structural proteins comprise the viral core [3]. Based on serology and the nucleotide sequence of the VP6 gene, rotavirus is divided into 9 groups or species (A to I). Rotavirus groups A, B, C, E, and H can all infect pigs, but RVA is the most common rotavirus associated with diarrhea in humans and animals [4, 5].

Currently, there are several commercially licensed rotavirus vaccines. The monovalent rotavirus vaccine Rotarix (comprising of the human G1P[8] strain) and the pentavalent RotaTeq (bovine-human rotavirus reassortants containing G1, G2, G3, G4, G6, P[8], P[7]) are routinely administered in industrialized countries as part of the universal childhood vaccination [6]. They have proven effective in preventing rotavirus-associated severe gastroenteritis [7]. Vaccination on swine farms with ProSystems Rota (consisting of porcine RVA strains like Gottfried G4[P6] and OSU G5P[7]) has also been implemented in some countries as part of livestock management towards blunting the transmission of RVA. Even so, vaccinating pig herds do not completely prevent RVA infection [2, 8]. Moreover, studies on the vaccine efficacy in the field and the availability of these vaccines are limited in many Asian countries including Thailand.

The Rotavirus Classification Working Group (RCWG) has assigned rotavirus genotypes of the 11 gene segments encoding VP7-VP4-VP6-VP1-VP2-VP3-NSP1-NSP2-NSP3-NSP4-NSP5, which corresponds to genotype designation Gx-P[x]-Ix-Rx-Cx-Mx-Ax-Nx-Tx-Ex-Hx, respectively[9, 10]. Genotypes G1-G4, G9, G12 (VP7) and P[4], P[6], P[8] (VP4) are mainly identified in human RVA, while genotypes G3-G5, G9, G11 and P[5]-P[7], P[23], P[28] are typical in infected pigs [2, 9]. Nevertheless, some genotypes such as G3, G4, G9 and P[6], P[19] frequently infect both pig and human [11].

RVA surveillance in Thailand is performed regionally and previously shows frequent combinations of G4P[6], G4P[19], G3P[19], G3P[23], G9P[19], G9P[23] [12–17]. We previously examined the prevalence of RVA on Thai swine farms and found that a significant number of RVA infection occurs in piglets [18]. As detailed knowledge of RVA genome constellation can better facilitate the understanding of rotavirus reassortment patterns and evolution, we now characterize the genetic diversity of 24 Thai porcine RVA strains previously identified between 2011 and 2016 for genotypic distribution, gene patterns, and the phylogenetic relationship among these strains compared to RVA strains in the vaccine.

## Materials and methods

### Samples

This study was approved by the Institutional Animal Care and Use Committee (IACUC number 1731020) and Institutional Biosafety Committee (IBC 1731008) of Chulalongkorn University. Twenty-four strains of RVA identified from feces and small intestinal contents (from the duodenum and upper part of jejunum) from animals with gastrointestinal illness (piglets before weaning, at weaning, nursery, finisher, pregnant and lactating sows) for which genome sequencing were successful were included in this study. These samples were randomly selected from among the RVA-positive samples from commercial pig herds in different provinces throughout Thailand identified between August 2011 to August 2016 [18]. Not all samples were submitted with information on the animal vaccination history and detailed clinical severity.

### Viral nucleic acid detection and sequencing

Samples were prepared as 10% (w/v) suspension with sterile phosphate buffered saline, centrifuged at 3,000 x g for 20 minutes, and supernatants were collected. Viral RNA was extracted using viral RNA purification kit (GeneAll, Seoul, Korea) according to manufacturer’s instructions. Samples were initially tested for RVA VP7 gene using SuperScript III One-Step RT-PCR with Platinum Taq DNA polymerase (Invitrogen, Carlsbad, CA). Cycling parameters were reverse transcription at 48°C for 45 minutes, initial denaturation at 95°C for 2 minutes, 35 cycles of denaturation at 94°C for 30 seconds, annealing at 55°C for 30 seconds, extension at 72°C for 90 seconds, and final extension at 72°C for 5 minutes. VP7-positive samples were subsequently subjected to partial amplification of gene segments encoding VP1, VP2, VP3, VP4, VP6, NSP1, NSP2, NSP3, NSP4, and NSP5 (S1 Table). PCR amplicons were resolved and purified using agarose gel electrophoresis. Nucleotide sequences were determined by Sanger sequencing, analyzed using SeqMan, and deposited in the GenBank database (accession numbers shown in S2 Table).

### Sequence and phylogenetic analyses

Strain genotypes were determined using the RotaC v2.0 automated genotyping tool [19]. Sequence alignments were performed using Clustral X v2.0.11 and reference sequences available from the GenBank database. Phylogenetic trees were constructed using MEGA6 software with the maximum likelihood method and 1,000 replicates [20]. Bootstrap values ≥80% were considered significant.

The deduced amino acid sequences from the Thai RVA strains were compared to those of the RVA strains in the porcine RVA vaccine and reference strains. Sequence identity in percentage was from amino acid comparison unless noted otherwise. For genotype G3, G4, G5, and G9, strain A131 (accession no. L35055), Gottfried (X06759), OSU (X04613), and A2 (AB180971) were used, respectively. The amino acid residue at position 291 was not analyzed because the sequence of the VP7 PCR product did not include this residue. For genotype P[6], P[13], P[19], and P[23], reference strains were Gottfried (M33516), HP140 (DQ003291), 4F (L10359), CMP48/08 (HQ268847), respectively. For genotype I1 and I5, Wa (K02086) and YM (X69487) served as reference strains. Specific reference strains were chosen based on recommendations by the RCWG and when their nucleotide sequences encompassed the same region as the Thai strains in this study [9, 10].

## Results

### G, P, and I genotypes

**Fig 1:**
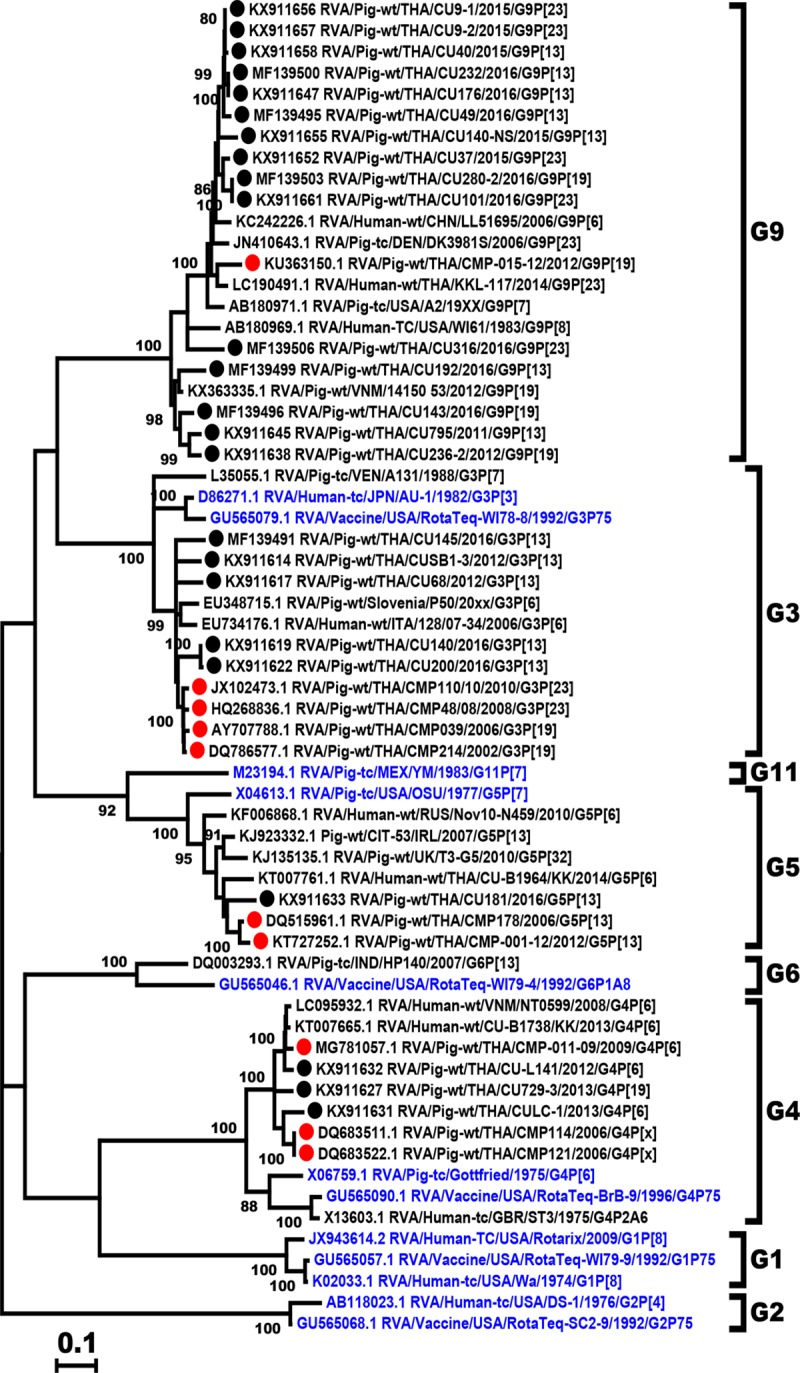
Phylogenetic analysis of the RVA VP7 gene. The nucleotide sequences of the Thai strains (black dotted) were compared to those of previous Thai porcine RVA strains (red dotted), the RVA reference and vaccine strains (blue).

The characterization of 24 RVA-positive samples from Thai swine obtained between 2011 and 2016 showed a predominance of G9 (62.5%, 15/24), followed by G3 (20.8%, 5/24), G4 (12.5%, 3/24), and G5 (4.2%, 1/24) (Fig 1). These Thai strains shared 82.5–100% amino acid identity to one another and 82.4–93.5% amino acid identity to the A2 reference strain. Several strains showed identical nucleotide sequences (RVA/Pig-wt/THA/CU101/2016/G9P[23] and RVA/Pig-wt/THA/CU280-2/2016/G9P[19], and RVA/Pig-wt/THA/CU176/2016/G9P[13] and RVA/Pig-wt/THA/CU232/2016/G9P[13]), all of which were identified in 2016 and were from two adjacent provinces. Of the Thai G4 strains (3/24), they were more closely related to RVA strains of recent years (92.2–93.7% amino acid identity) than to the prototypic porcine RVA strain Gottfried (80.8–82.8% identity). The 5 Thai G3 strains shared 88.7–99.6% identity and were similar to a Thai porcine strain (RVA/Pig-wt/THA/CMP39/2000/G3P[19]) previously identified as far back as 2000 (91.4–93.3% identity). The only one Thai G5 strain in this study (RVA/Pig-wt/THA/CU181/2016/G5P[13]) was also similar to a previously described Thai porcine strain (RVA/Pig-wt/THA/CMP178/2006/G5P[13] and RVA/Pig-wt/THA/CMP-001-12/2012/G5P[13] greater than 90%) compared to the prototypic porcine RVA strain OSU (81.8% identity).

**Fig 2:**
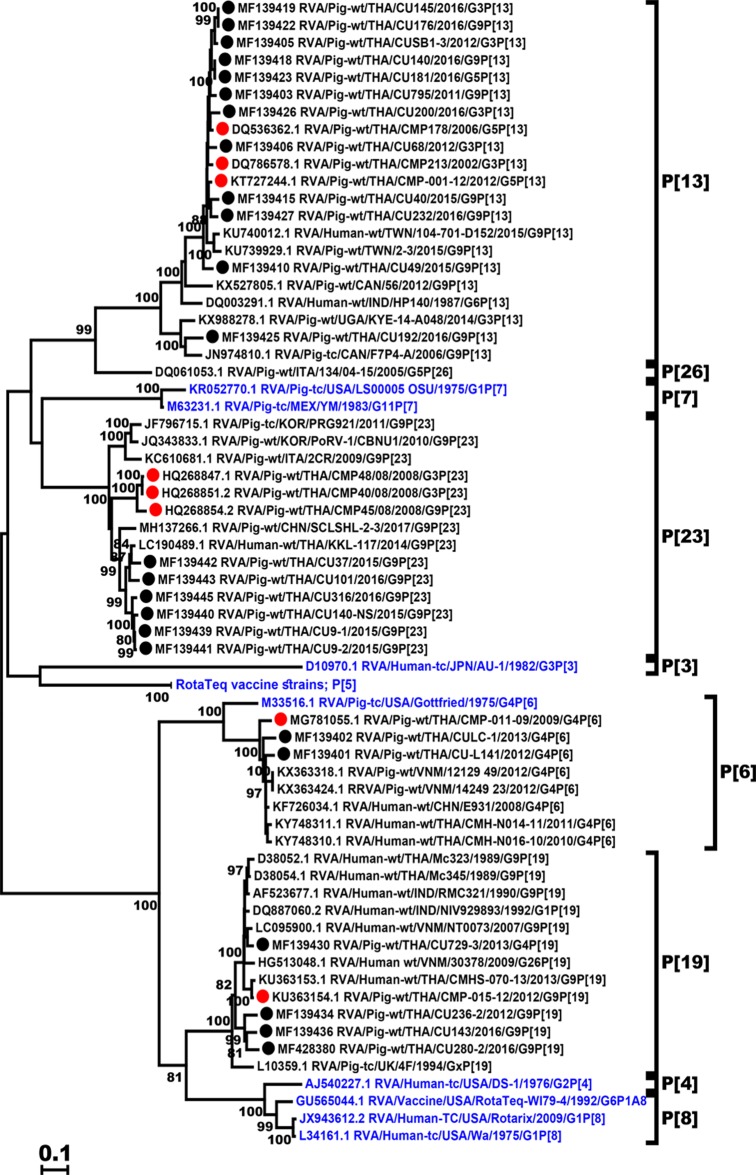
Phylogenetic analysis of the RVA VP4 gene. The nucleotide sequences of the Thai strains (dotted) were compared to those of previous Thai porcine RVA strains (red dotted), the RVA reference and vaccine strains (blue).

Four P genotypes were identified in our study, of which P[13] was predominant (50%, 12/24), followed by P[23] (25%, 6/24), P[19] (16.6%, 4/24), and P[6] (8.4%, 2/24). All Thai P[13] strains except RVA/Pig-wt/THA/CU192/2016/G9P[13] clustered in the same genetic group (Fig 2). Two Thai P[13] strains, RVA/Pig-wt/THA/CU140/2016/G9P[13] and RVA/Pig-wt/THA/CU181/2016/G5P[13], were identified in different provinces but showed identical nucleotide sequences. Interestingly, all Thai P[23] strains in this study shared 93–95.6% amino acid identity to RVA/Human-wt/THA/KKL-117/2014/G9P[23] previously identified in an infant with diarrhea in Thailand. The Thai P[19] strains shared 84.4–86.7% identity among each other and 87.5–96.4% identity with the reference strain RMC321. The Thai P[6] strains (RVA/Pig-wt/THA/CU-L141/2012/G4P[6] and RVA/Pig-wt/THA/CULC-1/2013/G4P[6]) showed closer genetic relatedness to an RVA strain identified from a patient (E931, 94–96.2% identity) than to Gottfried (75.8–76.4%).

**Fig 3:**
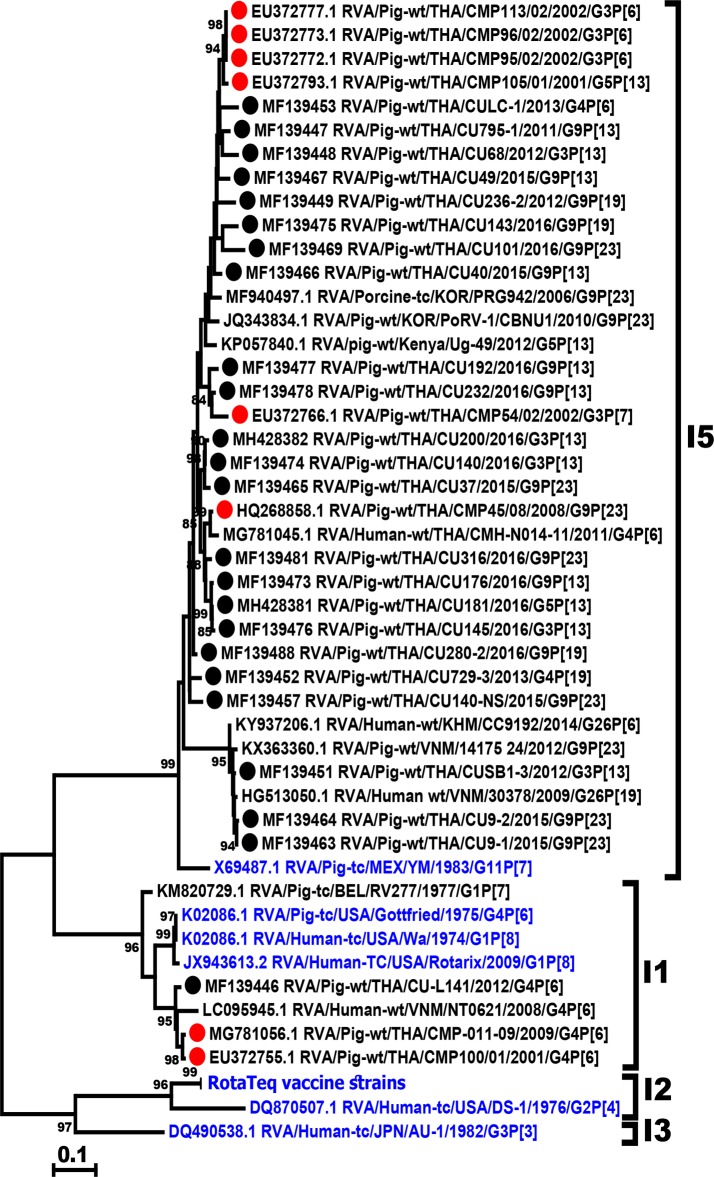
Phylogenetic analysis of the RVA VP6 gene. The nucleotide sequences of the Thai strains (dotted) were compared to those of previous Thai porcine RVA strains (red dotted), the RVA reference and vaccine strains (blue).

Overall, the dominant G and P combinations were G9P[13] and G9P[23] (n = 6 each), followed by G3P[13] (n = 5), G9P[19] (n = 3), G4P[6] (n = 2), G4P[19] and G5P[13] (n = 1 each) (Table 1). There were 23 strains of I5 genotype (88–95.3% amino acid identity), which clustered with the YM porcine reference strain (Fig 3). Interestingly, one Thai RVA strain (RVA/Pig-wt/CU-L141/2012/G4P[6]) belonged to I1 genotype along with Gottfried and Wa strains.

**Table 1:** The genome constellation of 24 Thai porcine strains.

Strain	Age	Sample	L	VP7	VP4	VP6	VP1	VP2	VP3	NSP1	NSP2	NSP3	NSP4	NSP5
**RVA/Pig-wt/THA/CU68/2012/G3P[13]**	3 d	s.i.	NP	G3	P[13]	I5	R1	C1	M1	A8	N1	T1	E1	H1
**RVA/Pig-wt/THA/CUSB1-3/2012/G3P[13]**	6 wk	feces	SB	G3	P[13]	I5	R1	C1	M1	A8	N1	T1	E1	H1
**RVA/Pig-wt/THA/CU145/2016/G3P[13]**	5 wk	feces	KB	G3	P[13]	I5	R1	C1	M1	A8	N1	T1	E1	H1
**RVA/Pig-wt/THA/CU140/2016/G3P[13]**	6 wk	feces	RB	G3	P[13]	I5	R1	C1	M1	A8	N1	T1	E1	H1
**RVA/Pig-wt/THA/CU200/2016/G3P[13]**	6 wk	feces	RB	G3	P[13]	I5	R1	C1	M1	A8	N1	T1	E1	H1
**RVA/Pig-wt/THA/CU-L141/2012/G4P[6]**	2 wk	s.i.	RB	G4	P[6]	I1	R1	C1	M1	A8	N1	T1	E1	H1
**RVA/Pig-wt/THA/CULC-1/2013/G4P[6]**	1 wk	feces	NP	G4	P[6]	I5	R1	C1	M1	A8	N1	T1	E1	H1
**RVA/Pig-wt/THA/CU729-3/2013/G4P[19]**	2 wk	feces	RB	G4	P[19]	I5	R1	C1	M1	A8	N1	T7	E1	H1
**RVA/Pig-wt/THA/CU181/2016/G5P[13]**	2 wk	feces	SP	G5	P[13]	I5	R1	-	M1	A8	N1	T1	E1	H1
**RVA/Pig-wt/THA/CU795/2011/G9P[13]**	5 d	s.i.	NP	G9	P[13]	I5	R1	C1	M1	A8	N1	T1	E1	H1
**RVA/Pig-wt/THA/CU40/2015/G9P[13]**	6 wk	feces	CB	G9	P[13]	I5	R1	-	M1	A8	N1	T1	E1	H1
**RVA/Pig-wt/THA/CU49/2015/G9P[13]**	3 wk	feces	CB	G9	P[13]	I5	R1	-	M1	A8	N1	T1	E1	H1
**RVA/Pig-wt/THA/CU176/2016/G9P[13]**	4 wk	feces	KB	G9	P[13]	I5	R1	C1	M1	A8	N1	T1	E1	H1
**RVA/Pig-wt/THA/CU232/2016/G9P[13]**	6 wk	feces	RB	G9	P[13]	I5	R1	C1	M1	A8	N1	T1	E1	H1
**RVA/Pig-wt/THA/CU192/2016/G9P[13]**	6 wk	feces	RB	G9	P[13]	I5	R1	C1	M1	A8	N1	T7	E1	H1
**RVA/Pig-wt/THA/CU236-2/2012/G9P[19]**	2 wk	feces	RB	G9	P[19]	I5	R1	C1	M1	A8	N1	T1	E1	H1
**RVA/Pig-wt/THA/CU280-2/2016/G9P[19]**	4 wk	feces	RB	G9	P[19]	I5	R1	C1	M1	A8	N1	T1	E1	H1
**RVA/Pig-wt/THA/CU143/2016/G9P[19]**	6 wk	feces	KB	G9	P[19]	I5	R1	C1	M1	A8	N1	T7	E9	H1
**RVA/Pig-wt/THA/CU9-1/2015/G9P[23]**	4 wk	s.i.	CB	G9	P[23]	I5	R1	C1	M1	A8	N1	T1	E1	H1
**RVA/Pig-wt/THA/CU9-2/2015/G9P[23]**	4 wk	s.i.	CB	G9	P[23]	I5	R1	C1	M1	A8	N1	T1	E1	H1
**RVA/Pig-wt/THA/CU140-NS/2015/G9P[23]**	6 wk	feces	CB	G9	P[23]	I5	R1	C1	M1	A8	N1	T1	E1	H1
**RVA/Pig-wt/THA/CU37/2015/G9P[23]**	10 d	feces	RB	G9	P[23]	I5	R1	C1	M1	A8	N1	T1	E1	H1
**RVA/Pig-wt/THA/CU101/2016/G9P[23]**	5 wk	feces	RB	G9	P[23]	I5	R1	C1	M1	A8	N1	T1	E1	H1
**RVA/Pig-wt/THA/CU316/2016/G9P[23]**	24 d	feces	NR	G9	P[23]	I5	R1	C1	M1	A8	N1	T1	E1	H1

d, day; wk, week; s.i., small intestine; L, farm location.

CB, Chon Buri; KB, Kanchanaburi; NP, Nakhon Pathom; NR, Nakhon Ratchasima

RB, Ratchaburi; SB, Saraburi; SP, Suphan Buri.

### Analysis of other genotypes

**Fig 4:**
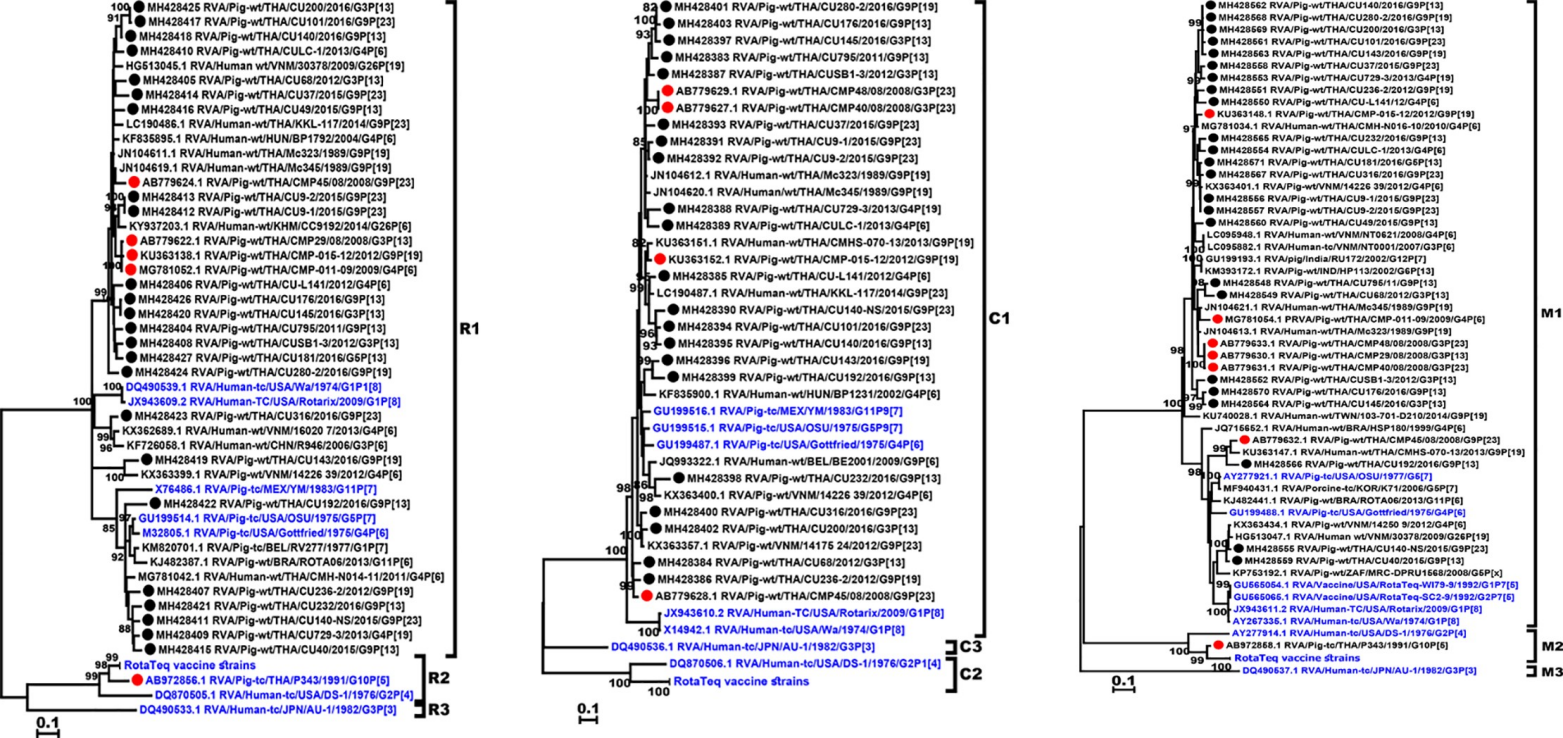
Phylogenetic analysis of the RVA VP1, VP2, and VP3 genes. The nucleotide sequences of the Thai strains (dotted) were compared to those of previous Thai porcine RVA strains (red dotted), the RVA reference and vaccine strains (blue).

The majority of the 24 Thai porcine RVA strains demonstrated a conserved internal genotype constellation of R1-C1-M1-A8-N1-T1-E1-H1. All Thai strains in our study belonged to the R1 genotype. The C genotypes were not generated from 3 strains due to insufficient PCR amplicon, but the remaining 21 strains were C1 genotype and shared 82–86% amino acid identity (86.2–92.8% identity with Gottfried, OSU, and YM strains). Among the Thai M1 genotype, only 3 strains clustered closely with Gottfried (81.6–84.9% identity), OSU (81.8–87.7% identity), and Wa (82.1–85% identity), while the rest clustered in a separate subgroup (Fig 4).

**Fig 5:**
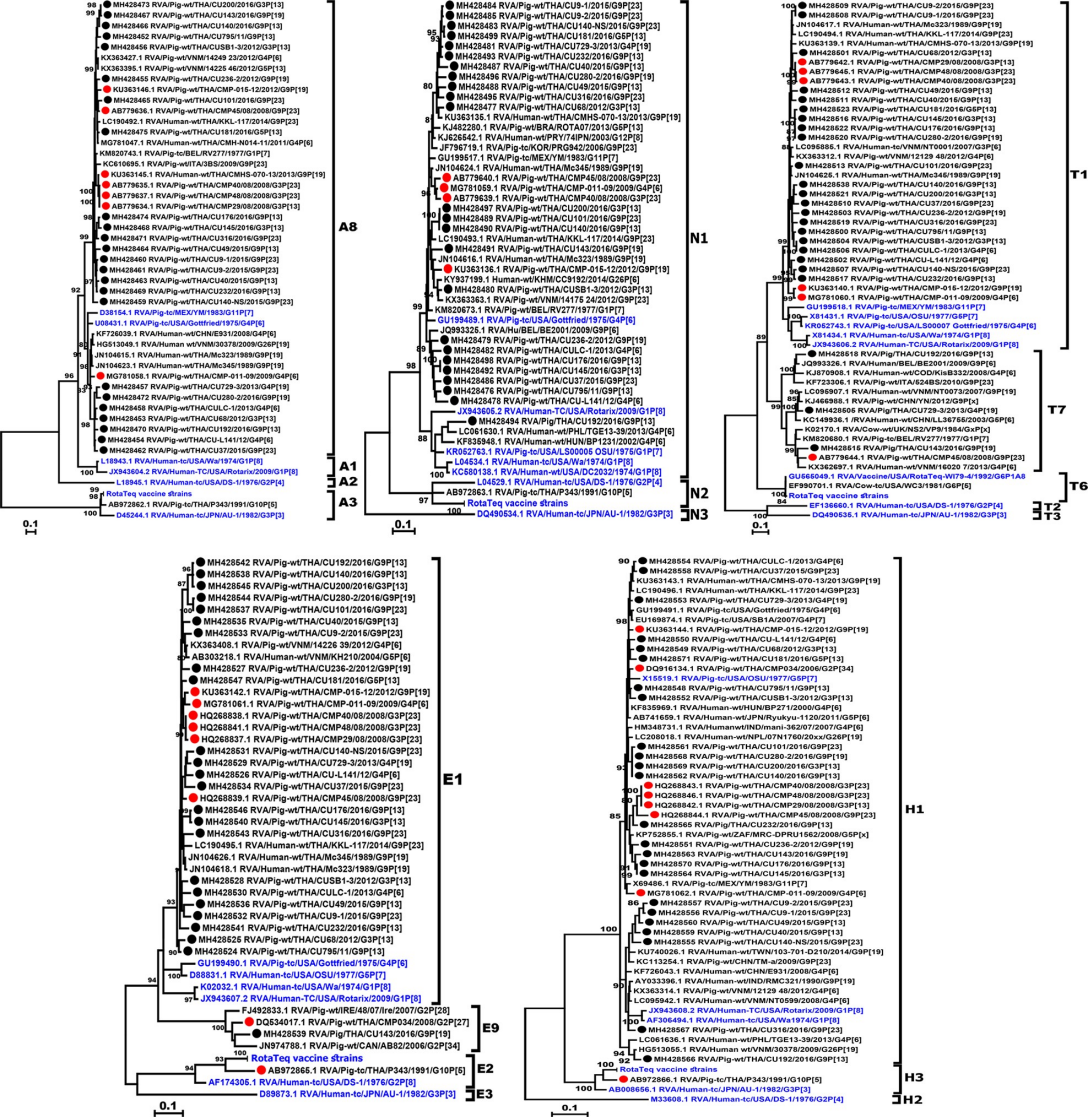
Phylogenetic analysis of the RVA non-structural genes. The nucleotide sequences of the Thai strains (dotted) were compared to those of previous Thai porcine RVA strains (red dotted), the RVA reference and vaccine strains (blue).

Analysis of the non-structural protein genes showed all strains were A8 genotype (Fig 5). Although seven of the Thai strains clustered with Gottfried, the majority comprised a separate branch containing relatively more recent RVA strains. Collectively, the Thai strains in these two lineages shared 78.4–85.8% amino acid identity. Most Thai N1 strains (n = 23) clustered with Gottfried, but one strain (RVA/Pig-wt/CU192/2016/G9P[13]) clustered with Wa and OSU. For T genotype, most Thai strains (n = 21) grouped with Gottfried, OSU, and Wa in the T1 cluster. The majority of the E genotype (n = 23) strains was E1 for which both the human and porcine RVA vaccine strains belonged. One E9 genotype (RVA/Pig-wt/CU143/2016/G9P[19]) was the only outlier strain and clustered with the reference strain RVA/Pig-wt/THA/CMP034/2008/G2P[27] previously identified in piglet on a Thai farm. Three Thai H1 genotype strains were identical in nucleotide sequences (RVA/Pig-wt/CU140/2016/G9P[13], RVA/Pig-wt/CU200/2016/G3P[13], and RVA/Pig-wt/CU280-2/2016/G9P[19]), which was not surprising since these were farm samples from the same province.

Taken together, the characterization of all 11 segments of RVA from 24 strains identified in Thailand over a 5-year period revealed a dominance of the constellation Gx-P[x]-I5-R1-C1-M1-A8-N1-T1-E1-H1. With the exception of I and A genotypes, this pattern resembles the prototypic RVA strain of Wa lineage (which possesses I1 and A1).

### Comparison of the antigenic epitopes on VP7 between the Thai porcine and the vaccine strains

**Fig 6:**
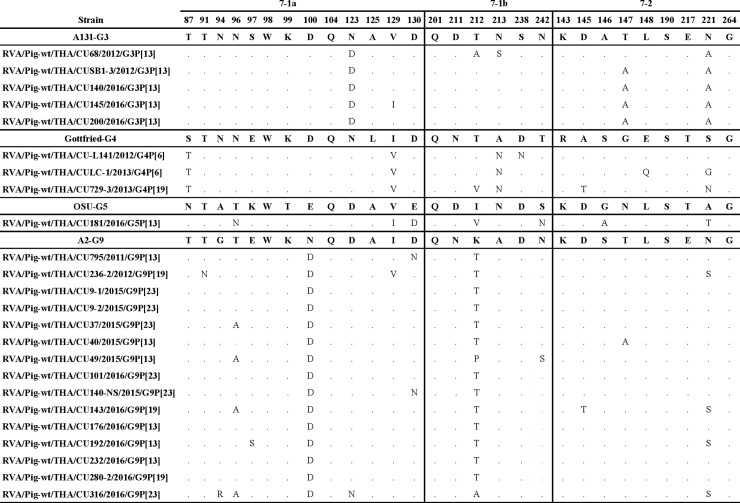
Residue differences in the VP7 antigenic regions between the Thai strains and the RVA reference/vaccine strains. Residue positions for each region are numbered. Identical residues are indicated by dots.

Amino acid variance on the antigenic epitopes of VP7 (namely region 7-1a, 7-1b and 7–2) among circulating RVA strains can influence the effectiveness of the vaccine used in the field. To determine residue differences on these important epitopes seen on the 24 Thai RVA strains, we compared them to Gottfried, OSU, and the other reference RVA strains (Fig 6). The Thai G3 strains demonstrated conservative amino acid change at N123D, and less so at T147A and N221A, compared to prototypic A131 strain. Comparison of the three Thai G4 strains to Gottfried showed common changes at S87T, I129V and A213N. Strain RVA/Pig-wt/THA/CU729-3/2013/G4P[19] possessed six residue changes, the most of any G4 strains. Comparison with OSU showed that the only Thai G5 strain displayed changes at T96N, V129I, E130D, I212V, S242N, G146A, and A221T. For most Thai G9 strains, they differed from the reference strain at N100D and K212T, with RVA/Pig-wt/CU316/2016/G9P[23] possessing the maximum of six residue variance.

## Discussion

Awareness of RVA infection on Thai swine farms are currently insufficient to appreciate the magnitude of viral circulation and the emergence of potentially novel viral reassortants. To better understand the genetic and antigenic relationship of circulating porcine RVA within Thailand and in comparison to the global and vaccine strains, we characterized the genome constellation of 24 Thai RVA strains from among the RVA-positive samples in our previous study [18]. In addition to G/P genotyping, the analysis of near-complete and partial gene regions of the non-G/P segments revealed inter-species RVA reassortants and the genetic diversity of RVA circulating on medium-to-large commercial Thai swine farms (S1 Fig).

Genotypes G5, I5, A1/A8, T1, and E1 are typically identified with porcine RVA, while T7 and E9 genotypes are more rare [21, 22]. Surprisingly, only one Thai strain in this study was G5, and genotype A1 was absent among our strains. While G3P[13] is relatively rare in the Americas, one in five Thai strains belonged to this genotype. We also identified two P[6] genotype strains, which was not found in RVA-infected Canadian pigs [23]. RVA genotype P[19] in combination with G1 and G3 is typically associated with human, while in combination with G4, G5, and G9 it is associated with porcine infection [16, 23–26]. In this study, we identified one G4P[19] and 3 G9P[19] strains, both of which were first detected in Thailand in 2009 and were phylogenetically more related to RVA derived from humans than from pigs [17]. Moreover, there were six strains of reportedly rare genotype G9P[23] from samples derived from various non-adjacent provinces between 2015 and 2016, which suggests that their infection is now widespread [13, 27].

Although several Thai strains in this study resembled the genome constellation of the prototypic Gottfried (Gx-P[x]-I1-R1-C1-M1-A8-N1-T1-E1-H1), the phylogenetic analysis of the nucleotide sequences showed notable strain differences. For example, a Thai RVA strain (RVA/Pig-wt/THA/CU-L141/12/G4P[6]) with human-like G genotype of constellation G4-P[6]-I1-R1-C1-M1-A8-N1-T1-E1-H1 was identified from a two-week-old piglet. In addition, variants of constellation G4-P[6]-I5-R1-C1-M1-A8-N1-T1-E1-H1 (strain RVA/Pig-wt/THA/CULC-1/2013/G4P[6]), G4-P[19]-I5-R1-C1-M1-A8-N1-T7-E1-H1 (strain RVA/Pig-wt/THA/CU729-3/2013/G4P[19]), G9-P[13]-I5-R1-C1-M1-A8-N1-T7-E1-H1 (strain RVA/Pig-wt/THA/CU192/2016/G9P[13]), and G9-P[19]-I5-R1-C1-M1-A8-N1-T7-E9-H1 (strain RVA/Pig-wt/THA/CU143/2016/G9P[19]) strongly suggest RVA genome reassortment during RVA co-infection and inter-species transmission. Furthermore, the G9P[19] and G9[P23] combination identified in this study were previously described in Thai children with diarrhea and on swine farms in northern Thailand and reportedly possessed identical genome constellations consistent with possible zoonotic transmission [26, 28]. The fact that our study also identified these genotypes in different regional pig farms of Thailand around the same time as these reports suggests that these strains were not restricted to northern Thailand and may have been circulating in the country over the past decade. Moreover, the recent circulating porcine RVA within Thailand clustered into the same major branch of all segments and also shared high genetic relatedness to previous Thai RVA strains identified since 2008.

Our study found that G3, G4, G5 and G9 genotypes possessed limited amino acid differences among the Thai and the vaccine strains. The amino acid identity of the Thai G4 strains was 80.8–82.8% compared to Gottfried, which was less than between the Thai G9 strains and A2 (82.4–93.5%). Although VP7 antigenic epitopes are critical to induce neutralizing antibody against rotavirus infection, antigenic epitopes on VP4 protein is also involved [29]. Characterization of the latter among the porcine Thai and the vaccine strains were not included because there were no P[7] in this study and the two P[6] strains were too few to make any comparison meaningful.

The licensed porcine RVA vaccine such as the ProSystems ROTA for young piglet is not available on Thai swine farms and is not routinely administered to animals even though RVA infection ranks second only to porcine enteric disease virus infection in this region [18]. None of the pigs in which our samples were derived were vaccinated for RVA, therefore the genotypes identified in this study were probably naturally circulating and unlikely to have resulted from vaccine-escaped variants. In addition to good husbandry practice, pig farmers often feed pregnant sows with either minced intestinal content from infected piglets or diluted sow feces as a low-cost way of eliciting immunity against porcine enteric disease virus infections within the Thai pig herds. This practice may inadvertently foster periodic outbreaks of RVA, which causes recurrent infection within the same herd and perpetuates its circulation on pig farms. This might be a reason why identical sequences were found in several gene segments of Thai porcine RVA strains despite different regions and time of collection. For instance, RVA/Pig-wt/CUSB1-3/2012/G3P[13] shared identical NSP3 amino acid sequence to RVA/Pig-wt/CULC-1/2013/G4P[6] even though they were collected from non-adjacent provinces and different years. This may simply be due to coincidence, or to animal transportation throughout the region of commercial pig farms in spreading RVA. Identical amino acid sequences encoded by the NSP5 gene were also found among 3 strains (RVA/Pig-wt/CU140/2016/G9P[13], RVA/Pig-wt/CU200/2016/G3P[13], and RVA/Pig-wt/CU280-2/2016/G9P[19]). Although these strains were collected from herds in the same province, pigs were infected in February, April, and July 2016, respectively. Non-structural proteins are not under as much immune pressure compared to capsid proteins, therefore it was not surprising to find more similarities occurring in these gene segments. Nevertheless, RVA strains with identical genome constellation reappearing year after year, and on farms located in different provinces, further underscore the problem of transporting animals across farms in exacerbating RVA transmission and the endemicity of RVA in pig herds on commercial swine farms.

Pigs are natural reservoir for RVA transmission to human, as close contact between farm animals and pig handlers enable atypical RVA reassortants to emerge [30–32]. Human RVA strains RVA/Human-wt/THA/Mc323/1989/G9P[19] and RVA/Human-wt/THA/Mc345/1989/G9P[19] isolated in 1989, CMH-S070-13/2013/G9P[19], and KKL117/2014/G9P[23] all historically derived from Thai patients are phylogenetically close to the Thai porcine RVA strains in this study and shared >90% amino acid identity in the majority of the non-G/P genotypes. In fact, previous studies have concluded that these RVA strains were likely of porcine origin [25, 26, 28]. Thus, surveillance of porcine RVA has important implications for human health.

This study has several limitations. Identification of additional genome constellations may have been missed as only 24 strains were analyzed in this study and none of which were from small family-owned farms. The samples submitted to us for analysis may have potentially been biased as farm participations were not universally represented. More samples in which the sequence information were derived were from western provinces of Thailand where swine farms are predominantly located. Although characterizing amino acid differences in the antigenic epitope regions provided important clues to the potential vaccine effectiveness, analysis of the residue changes in the circulating RVA alone may not accurately predict potential vaccine escapes. In summary, continued epidemiological studies of RVA on swine farms will be important in the justification of animal vaccination and the inclusion of additional strains in the development of improved vaccines.

## Supporting information

S1 Table Nucleotide primers used in the amplification of RVA genes. (DOCX)

S2 Table Accession numbers of Thai RVA strains used in this study. (DOCX)

S1 Fig Thailand map showing G/P genotype distributions of Thai porcine RVA strains in this study. (TIF)
