# Supplementary material for: Genome constellations of 24 porcine rotavirus group A strains circulating on commercial Thai swine farms between 2011 and 2016
Source: PLoS One. 2019 Jan 23;14(1):e0211002. doi: 10.1371/journal.pone.0211002 (PMC6343967; doi:10.1371/journal.pone.0211002)
Supplement: S1 Table — (DOCX) [file pone.0211002.s001.docx]

**S1 Table. Nucleotide primers used in the amplification of RVA genes.**

| **Primers** | **Sequences (5' to 3')** | **Location** | **Annealing** | **Amplicon** |
| --- | --- | --- | --- | --- |
|  |  |  |  |  |
| **VP7 gene** | CGGTTAGCTCCTTTTAATGT | 33-52 | 55°C | 891 bp |
| (AB176677.1) | CATTTCTTCCAATTTACTCGC | 903-924 |  |  |
| **VP4 gene** | GGCTATAAATGGCTTCGCTC | 1-21 | 52 °C | 1074 bp |
| (Winiarczyk 2002) | AATGCTTGTGAATCATCCCAG | 1074-1094 |  |  |
| **VP6 gene** | GTCTTCGACATGGAAGTTC | 15-33 | 46°C | 633 bp |
| (AB779621) | AAACTGYTGAATRTTTGCTGG | 627-649 |  |  |
| **VP1 gene** | GGAAGTAYAATCTAATCTTG | 23-42 | 42°C | 1075 bp |
| (DQ490539.1) | TACATYTCRYYATCHACATC | 1078-1098 |  |  |
| **VP2 gene** | TTTCCDACHATGCCDGTTGA | 1598-1617 | 44°C | 1097 bp |
| (JX406748.1) | GGCGTYTACARYTCRTTCAT | 2675-2694 |  |  |
| **VP3 gene** | GGCTWTTAAAGCARAYTAGTAG | 1-23 | 50°C | 630 bp |
| (Silva 2015 ) | TTTRTCTCTRAAYACACGATTTGA | 608-631 |  |  |
| **NSP1 gene** | TTATGAAAAGTCTTGTGGAA | 9-28 | 46°C | 545 bp |
| (JX406751.1) | AAATGARAATGGDGTYTGATT | 533-553 |  |  |
| **NSP2 gene** | TTTTAAAGCGTCTCAGTCG | 4-22 | 48°C | 727 bp |
| (JX406754.1) | AACTCTRTAGTGACCTTTACC | 710-730 |  |  |
| **NSP3 gene** | TTTCAGTGGTTGTTGCTCA | 14-32 | 52°C | 995 bp |
| (JX406753.1) | AGAGGGTYAYGTGWAGATGG | 989-1008 |  |  |
| **NSP4 gene** | AAAGTTCTGTTCCGAGAGA | 19-27 | 52°C | 732 bp |
| (JX406756.1) | AGACCRTTCCTTCCATTAACG | 720-740 |  |  |
| **NSP5 gene** | GCTACAGTGATGTCTCTCAGC | 13-33 | 52°C | 580 bp |
| (GU199491.1) | TTGCGACTTGCTTCATCCTC | 579-592 |  |  |
|  |  |  |  |  |
| **D=**A/G/T; **H=**A/C/T; **R=**A/G; **Y=**C/T. | |  |  |  |

Winiarczyk S, Paul PS, Mummidi S, Panek R, Gradzki Z. Survey of porcine rotavirus G and P genotype in Poland and the United States using RT-PCR. J Vet Med B Infect Dis Vet Public Health. 2002;49(8):373-8.

Silva FD, Espinoza LR, Tonietti PO, Barbosa BR, Gregori F. Whole-genomic analysis of 12 porcine group A rotaviruses isolated from symptomatic piglets in Brazil during the years of 2012-2013. Infect Genet Evol. 2015;32:239-54.
