# Supplementary material for: Genome constellations of 24 porcine rotavirus group A strains circulating on commercial Thai swine farms between 2011 and 2016
Source: PLoS One. 2019 Jan 23;14(1):e0211002. doi: 10.1371/journal.pone.0211002 (PMC6343967; doi:10.1371/journal.pone.0211002)
Supplement: S2 Table — (DOCX) [file pone.0211002.s002.docx]

**S2 Table. Accession numbers of Thai RVA strains used in this study.**

|  | **Collection date** |  |  |  |  | **Accession number** |  |  |  |  |  |  |
| --- | --- | --- | --- | --- | --- | --- | --- | --- | --- | --- | --- | --- |
| **Strain name** |  | **VP7** | **VP4** | **VP6** | **VP1** | **VP2** | **VP3** | **NSP1** | **NSP2** | **NSP3** | **NSP4** | **NSP5** |
| **RVA/Pig-wt/THA/CU795/2011/G9P[13]** | 2011 | KX911645 | MF139403 | MF139447 | MH428404 | MH428383 | MH428428 | MH428452 | MH428476 | MH428500 | MH428524 | MH428548 |
| **RVA/Pig-wt/THA/CU68/2012/G3P[13]** | 2012 | KX911617 | MF139406 | MF139448 | MH428405 | MH428384 | MH428429 | MH428453 | MH428477 | MH428501 | MH428525 | MH428549 |
| **RVA/Pig-wt/THA/CU-L141/2012/G4P[6]** | 2012 | KX911632 | MF139401 | MF139446 | MH428406 | MH428385 | MH428430 | MH428454 | MH428478 | MH428502 | MH428526 | MH428550 |
| **RVA/Pig-wt/THA/CU236-2/2012/G9P[19]** | 2012 | KX911638 | MF139434 | MF139449 | MH428407 | MH428386 | MH428431 | MH428455 | MH428479 | MH428503 | MH428527 | MH428551 |
| **RVA/Pig-wt/THA/CUSB1-3/2012/G3P[13]** | 2012 | KX911614 | MF139405 | MF139451 | MH428408 | MH428387 | MH428432 | MH428456 | MH428480 | MH428504 | MH428528 | MH428552 |
| **RVA/Pig-wt/THA/CU729-3/2013/G4P[19]** | 2013 | KX911627 | MF139430 | MF139452 | MH428409 | MH428388 | MH428433 | MH428457 | MH428481 | MH428505 | MH428529 | MH428553 |
| **RVA/Pig-wt/THA/CULC-1/2013/G4P[6]** | 2013 | KX911631 | MF139402 | MF139453 | MH428410 | MH428389 | MH428434 | MH428458 | MH428482 | MH428506 | MH428530 | MH428554 |
| **RVA/Pig-wt/THA/CU140-NS/2015/G9P[23]** | 2015 | KX911655 | MF139440 | MF139457 | MH428411 | MH428390 | MH428435 | MH428459 | MH428483 | MH428507 | MH428531 | MH428555 |
| **RVA/Pig-wt/THA/CU9-1/2015/G9P[23]** | 2015 | KX911656 | MF139439 | MF139463 | MH428412 | MH428391 | MH428436 | MH428460 | MH428484 | MH428508 | MH428532 | MH428556 |
| **RVA/Pig-wt/THA/CU9-2/2015/G9P[23]** | 2015 | KX911657 | MF139441 | MF139464 | MH428413 | MH428392 | MH428437 | MH428461 | MH428485 | MH428509 | MH428533 | MH428557 |
| **RVA/Pig-wt/THA/CU37/2015/G9P[23]** | 2015 | KX911652 | MF139442 | MF139465 | MH428414 | MH428393 | MH428438 | MH428462 | MH428486 | MH428510 | MH428534 | MH428558 |
| **RVA/Pig-wt/THA/CU40/2015/G9P[13]** | 2015 | KX911658 | MF139415 | MF139466 | MH428415 | N/A | MH428439 | MH428463 | MH428487 | MH428511 | MH428535 | MH428559 |
| **RVA/Pig-wt/THA/CU49/2015/G9P[13]** | 2015 | MF139495 | MF139410 | MF139467 | MH428416 | N/A | MH428440 | MH428464 | MH428488 | MH428512 | MH428536 | MH428560 |
| **RVA/Pig-wt/THA/CU101/2016/G9P[23]** | 2016 | KX911661 | MF139443 | MF139469 | MH428417 | MH428394 | MH428441 | MH428465 | MH428489 | MH428513 | MH428537 | MH428561 |
| **RVA/Pig-wt/THA/CU140/2016/G9P[13]** | 2016 | KX911619 | MF139418 | MF139474 | MH428418 | MH428395 | MH428442 | MH428466 | MH428490 | MH428514 | MH428538 | MH428562 |
| **RVA/Pig-wt/THA/CU143/2016/G9P[19]** | 2016 | MF139496 | MF139436 | MF139475 | MH428419 | MH428396 | MH428443 | MH428467 | MH428491 | MH428515 | MH428539 | MH428563 |
| **RVA/Pig-wt/THA/CU145/2016/G3P[13]** | 2016 | MF139491 | MF139419 | MF139476 | MH428420 | MH428397 | MH428444 | MH428468 | MH428492 | MH428516 | MH428540 | MH428564 |
| **RVA/Pig-wt/THA/CU232/2016/G9P[13]** | 2016 | MF139500 | MF139427 | MF139478 | MH428421 | MH428398 | MH428445 | MH428469 | MH428493 | MH428517 | MH428541 | MH428565 |
| **RVA/Pig-wt/THA/CU192/2016/G9P[13]** | 2016 | MF139499 | MF139425 | MF139477 | MH428422 | MH428399 | MH428446 | MH428470 | MH428494 | MH428518 | MH428542 | MH428566 |
| **RVA/Pig-wt/THA/CU316/2016/G9P[23]** | 2016 | MF139506 | MF139445 | MF139481 | MH428423 | MH428400 | MH428447 | MH428471 | MH428495 | MH428519 | MH428543 | MH428567 |
| **RVA/Pig-wt/THA/CU280-2/2016/G9P[19]** | 2016 | MF139503 | MH428380 | MF139487 | MH428424 | MH428401 | MH428448 | MH428472 | MH428496 | MH428520 | MH428544 | MH428568 |
| **RVA/Pig-wt/THA/CU200/2016/G3P[13]** | 2016 | KX911622 | MF139426 | MH428382 | MH428425 | MH428402 | MH428449 | MH428473 | MH428497 | MH428521 | MH428545 | MH428569 |
| **RVA/Pig-wt/THA/CU176/2016/G9P[13]** | 2016 | KX911647 | MF139422 | MF139473 | MH428426 | MH428403 | MH428450 | MH428474 | MH428498 | MH428522 | MH428546 | MH428570 |
| **RVA/Pig-wt/THA/CU181/2016/G5P[13]** | 2016 | KX911633 | MF139423 | MH428381 | MH428427 | N/A | MH428451 | MH428475 | MH428499 | MH428523 | MH428547 | MH428571 |
