# Supplementary figures and images for: Genome constellations of 24 porcine rotavirus group A strains circulating on commercial Thai swine farms between 2011 and 2016
Source: PLoS One. 2019 Jan 23;14(1):e0211002. doi: 10.1371/journal.pone.0211002 (PMC6343967; doi:10.1371/journal.pone.0211002)

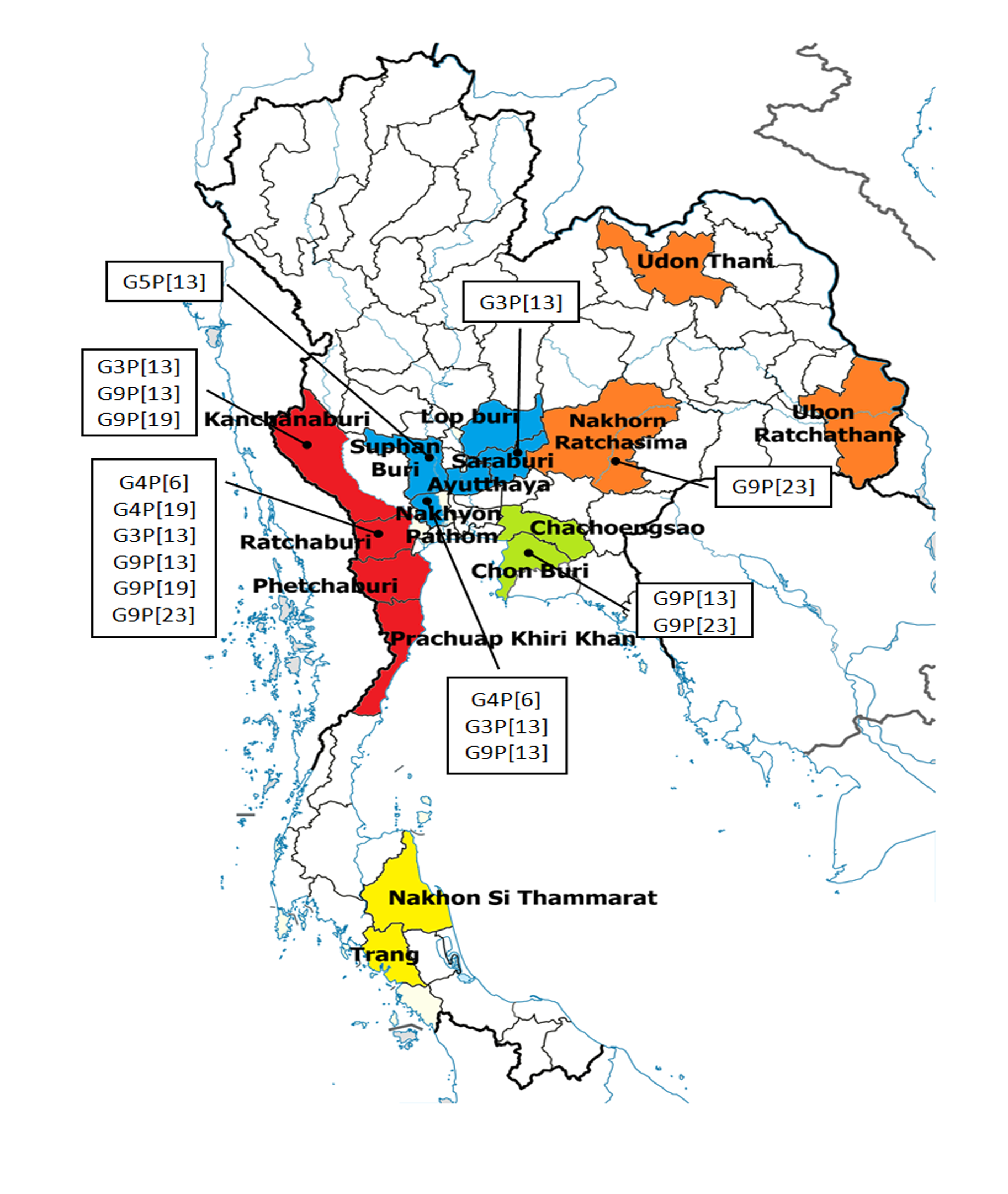

Supplement: S1 Fig — (TIF) [file pone.0211002.s003.tif]
